# Health risk factors and self-rated health among job-seekers

**DOI:** 10.1186/1471-2458-11-659

**Published:** 2011-08-19

**Authors:** Jennis Freyer-Adam, Beate Gaertner, Stefanie Tobschall, Ulrich John

**Affiliations:** 1 University Medicine Greifswald, Institute of Epidemiology and Social Medicine, Walther-Rathenau-Str. 48, 17475 Greifswald, Germany; 2 Charité University Medicine Berlin, Institute of Biometry and Clinical Epidemiology, Luisenstr. 65, 10117 Berlin, Germany; 3 Robert Koch Institute, Department of Epidemiology and Health Monitoring, General-Pape-Str. 62-66, 12101 Berlin, Germany

**Keywords:** addictive behavior, body mass index, health behavior, unemployment & health, health promotion

## Abstract

**Background:**

To determine a) proportions of behavior related health risk factors among job-seekers and b) to what extend these are related to self-rated health.

**Methods:**

Over 12 months, job-seekers were recruited at three job-agencies in northeastern Germany. Among all individuals eligible for study inclusion, 7,906 (79.8%) provided information on smoking, risky drinking, overweight/obesity (body mass index), fruit and vegetable intake, physical inactivity, illicit drug use, and self-rated health. Proportions and 95% confidence intervals stratified by gender, age and duration of unemployment were calculated. Multivariate logistic regression analyses predicting self-rated health were conducted.

**Results:**

The proportions of each health-risk factor were high, and 52.4% of the sample (53.4% male, 33.5 years mean age) had 3 or more health risk factors. Mostly, the proportions were particularly high among men and long-term unemployed individuals; e.g. 84.8% of the 18-24 year old long-term unemployed men were current smokers. Proportions of substance use related health risk factors were highest among the 18-24 year olds (e.g. risky drinking 28.7%), and proportions of health risk factors related to nutrition and physical inactivity were highest among the 40-64 year olds (e.g. overweight/obesity 65.4%). Depending on gender, all health risk factors and having 3 or more health risk factors were associated with lower self-rated health; odd ratios ranged between 1.2 for smoking (95% CI: 1.0-1.3) and 1.7 for overweight and physical inactivity (95% CI: 1.5-1.9).

**Conclusions:**

Prevention efforts to reduce health risk factors and to increase health among job-seekers are needed, and job agencies appear a feasible setting for their implementation.

## 1. Background

Unemployment attenuates social economic position differences in poor health [1]. Differences in health behaviors may explain this effect. For example, unemployed men are more often smokers than employed men, and unemployed individuals more often drink heavily and use illicit drugs [2,3]. Furthermore, long-term unemployed individuals are more often obese than stably employed individuals [4], and unemployed men and women spend less time with sports than employed men and women [5]. Consequently, e.g. in Germany 23% of the unemployed and 11% of the employed individuals describe their health as fair or poor; unemployed individuals have higher annual illness rates, spend about twice as many days hospitalized than employed individuals, and the risk of mortality increases with longer duration of unemployment [5].

Most studies investigating health risk behaviors and self-rated health of unemployed individuals have derived their data from general population surveys, and may be biased by low response rates. For the determination of health risk behaviors among job-seekers, it may be promising to contact job-seekers at job agencies where all job-seekers from a defined area may be reached. Only few studies have used such an approach, and these suggest that most individuals contacted at job-agencies are willing to give information on their health risk behaviors and to receive a minimal intervention [6,7].

The aim of our study was to determine proportions of individuals with health risk behaviors (current smoking, risky alcohol drinking, overweight, low fruit and vegetable intake, physical inactivity, illicit drug use) among a large sample of job-seekers recruited from different job agencies. We expected high proportions of individuals with health risk factors and high proportions of individuals with multiple health risk behaviors. The second aim was to investigate to what extend these health risk factors are associated with self-rated health. We assumed significant associations with lower self-rated health. Further, we wanted to derive conclusions regarding the suitability of job-agencies as a target setting to recruit job-seekers for purposes of health behavior change interventions.

## 2. Methods

The study was based on data collected as part of the randomized controlled Trial Of Proactive Alcohol interventions among job-Seekers (TOPAS, http://ClinicalTrials.gov Identifier: NCT01311245). The trial was conducted by the Research Collaboration on EARLy INTervention in health risk behaviors (EARLINT) in Western Pomerania, northeastern Germany. The local ethics committee of the Ernst-Moritz-Arndt-University of Greifswald approved the study.

### 2.1 Sample Recruitment

In Germany, the majority of unemployed individuals register at government or municipal owned job agencies. Registering at these job agencies is required for receiving unemployment compensation. Individuals threatened by job-loss (including those finishing education without having a job at hand or those below the minimal income limit) are also required to register at these job agencies. Each job-seeker who currently receives financial support or who is threatened by job loss has to contact her/his agent on a regular basis. All job-seekers, despite of their duration of unemployment (including those not yet unemployed) were included in the following study.

Between July 7, 2008 and July 10, 2009 three job agencies subsequently participated in the study. The recruitment of study participants at each job agency took between 16 and 19 weeks. During opening hours a total population screening was aspired. On each working day one or two study assistants approached all job-seekers who appeared in the waiting area to talk to a job agent. They were asked to participate in a screening regarding health behaviors; the inclusion criterion being individuals between 18 and 64 years old. Individuals cognitively or physically incapable, individuals already recruited for the study during an earlier visit, individuals with insufficient language or reading skills, and escorting individuals were excluded from the study. The participants answered questions provided by handheld computers.

Participation: A total of 26,178 visitors were registered by the staff of the study. Of these, 6,340 did not meet inclusion criteria (5,325 did not intend to talk to a job-agent, 909 had no waiting time, 106 were not 18-64 years old), and 9,925 were excluded from the screening (6,409 were already asked to participate during an earlier visit, 3,290 were escorting individuals, 200 had insufficient language/reading skills, 26 were cognitively or physically incapable). The remaining 9,913 visitors were eligible for the study. Of these, 7,920 (79.9%) responded to the screening, 1,552 (15.7%) declined to participate, and 441 (4.4%) did not participate due to other reasons (223 had no glasses at hand, 118 had no time, 67 dropped-out before responding to first item, 19 had trouble handling handheld, 14 were intoxicated). Of the screening respondents, 13 were excluded due to inconsistent data (e.g. an 18-year old female reported six children), and one had completely missing data due to technical problems, leaving a total sample of 7,906 participants (79.8%).

### 2.2 Measures

The screening contained items regarding socio-demographics, job-seeking status, health risk factors and self-rated health.

#### a) Socio-demographics

Gender and three further demographic variables were assessed. Age: To obtain three age groups with about equal numbers of subjects, 33% and 66% tertiles were calculated, resulting in: 18 to 24 years, 25 to 39 years, and 40 to 64 years. School education: Common German types of school education were assessed. For international comparability, these were categorized as: < 10 years, 10 to 11 years, and > 11 years of school (including those still in school). Marital status: was measured using one item with four response categories: single, married, divorced/separated, and widowed. Living in a steady partnership was assessed using one separate item with yes and no as response categories. Those married did not receive this item and were considered as living in a steady partnership.

#### b) Duration of unemployment

The duration of total life-time unemployment was assessed asking for the number of months or years unemployed altogether. Using 34% and 67% tertiles, three groups were obtained: non- or short-term unemployed (< 6 months), medium-term unemployed (6 to 24 months), and long-term unemployed (> 24 months).

#### c) Health risk factors

Tobacco smoking was assessed using the question "Are you a tobacco smoker currently?". Three response categories differentiated between current daily smoking, occasional smoking and non-smoking. Current occasional and daily smokers were considered smokers.

Risky drinking (e.g. > 12 g/> 24 g of pure alcohol per day for women/men [8]) was determined using the German adaptation of the first three items of the Alcohol Use Disorder Identification Test [AUDIT, 9]. The AUDIT-C, a common short form of the AUDIT, contains three items on alcohol consumption, and identifies individuals with risky drinking [10]. According to recommendations by Reinert and Allen [10], gender specific cut-off values of four for women and five for men were applied to determine risky drinking.

Overweight was assessed using the body mass index (BMI) obtained by self-reported weight and height in kg and cm, respectively. The obtained BMI (= kg/m^2^) was then categorized into four groups [11]: 1) underweight: BMI < 18.5, 2) normal weight: BMI 18.5 to < 25.0, 3) overweight: BMI 25.0 to < 30.0, and 4) obesity: BMI ≥ 30.0. It was further dichotomized: no overweight/obesity (BMI < 25.0) versus overweight/obesity (BMI ≥ 25.0).

Low fruit and vegetable intake was assessed using the single item „How many portions of fruit and vegetables do you normally eat per day?" and a six-point-rating scale: "none" to "5 and more". One portion was described as e.g. an apple, a little bowl of salad or a handful of vegetables (except potatoes). One or more glasses of fruit/vegetable juice (0, 2l) were to be counted as one portion in total. A fruit and vegetable intake of at least five portions a day is commonly recommended [12], and was used as the cut-off value in this study.

Physical inactivity was measured using two items to take into account different aspects. Every day physical activity was measured using the question „How many minutes per day do you spend walking or cycling, e.g. to do your (grocery) shopping, to go to school or to work?" and five response categories: < 5 minutes, 5 to 15 minutes, 15 to 30 minutes, 30 to 45 minutes, and > 45 minutes. Sports activity was measured using the question „Do you also do sports?" with six response categories: none, < 1 hour per week, 1 to 2 hours, 2 to 3 hours, 3 to 4 hours, and > 4 hours per week. Those individuals who reported < 30 minutes of every day physical activity and < 1 hour of additional sports per week were considered as physically inactive.

Illicit drug use was assessed according to the screening item of the CID-S [13] with a time span of 12 months „Have you used drugs such as hashish, ecstasy, cocaine or heroin more than 5 times in the past 12 months?". It included two response categories: yes and no.

The total number of health risk factors was calculated, with a maximum of six health risk factors. The number was then dichotomized: ≥ 3 health risk factors versus < 3 health risk factors.

#### d) Self-rated health

Self-rated health was assessed using the single item "Would you say your health in general is: excellent (1), very good (2), good (3), fair (4), poor (5)?". The item is known to be an independent predictor of mortality [14]. For the analyses, the responses were dichotomized: lower self-rated health versus better self-rated health.

### 2.3 Statistical Analyses

To determine proportions of individuals with a single health risk factor and with ≥ 3 health risk factors, percentages and 95% confidence intervals were calculated. These were stratified by gender, age, and duration of total life-time unemployment. These variables were entered as predictors in seven multivariate logistic regression analyses predicting each health risk factor and having ≥ 3 health risk factors. Differences between groups are reported when 95% confidence intervals between groups were not overlapping. To determine predictors of self-rated health, four multivariate logistic regression analyses were conducted, with socio-demographic variables entered as covariates. The first three regressions included all six health risk factors as predictors of self-rated health, separately for men and women, and for the total sample. The fourth regression included the total number of health risk factors as the only predictor of self-rated health among the total sample. Missing values were excluded list-wise. STATA SE 10 was applied [15].

## 3. Results

### 3.1 Sample Description

As depicted in Table 1, half of the total sample screened was male (53.4%) and the mean age was 33.5 years (SD = 12.5). Most of the participants were single (63.1%), and had 10 to 11 years of schooling (58.0%). The mean duration of total life-time unemployment was 29.2 months (SD = 41.2).

**Table 1:** Socio-demographics of the sample (n = 7,906)

Variables		N	%
Gender	Female	3,685	46.6
	Male	4,221	53.4
Age in years (mean, SD)		33.5	12.5
Age groups	18-24 years	2,599	33.1
	25-39 years	2,711	34.6
	40-64 years	2,537	32.3
Family status	Married	1,970	25.3
	Single	4,913	63.1
	Divorced/separated	808	10.4
	Widowed	90	1.2
Living in a steady relationship		4,792	61.7
School education	< 10 years	1,733	22.7
	10-11 years	4,415	58.0
	> 11 years	1,469	19.3
Life-time unemployment in months (mean, SD)		29.2	41.2
Life-time unemployment grouped	< 6 months*	2,675	35.2
	6-24 months	2,471	32.5
	> 24 months	2,447	32.2

*Notes: *N = number of cases, SD = standard deviation.

*including 648 job-seekers that have not yet been unemployed.

### 3.2 Tobacco smoking

Fifty-eight percent of the total sample were current smokers (Table 2). The logistic regression analysis revealed that gender (p < 0.001), age (p < 0.001) and duration of unemployment (p < 0.001) were significantly related to smoking. Except from the 18-24 year olds, smoking was more prevalent among men than among women. Smoking proportions were lowest among the 40-64 year olds (47.2%) and highest among the 18-24 year olds (64.9%). There were higher proportions of current smokers among the long-term unemployed than among the short-term unemployed. Overall, the highest proportions were found for long-term unemployed 18-24 year old women and men (80.2%, 84.8%).

**Table 2:** Proportions and 95% confidence intervals of tobacco smoking and risky alcohol drinking stratified by gender, age and months unemployed

	Tobacco smoking			Risky drinking		
	
	Total	Women	Men	Total	Women	Men
N	7,500	3,506	3,994	7,420	3,474	3,945
Total	57.7; 56.6-58.8	53.3; 51.6-55.0	61.6; 60.1-63.1	24.8; 23.8-25.8	16.5; 15.3-17.8	32.1; 30.6-33.6

18-24 years old	64.9; 63.0-66.8	63.5; 60.7-66.3	66.2; 63.5-68.7	28.7; 26.9-30.5	21.8; 19.5-24.3	35.0; 32.4-37.7
0-6 months	59.7; 57.2-62.2	60.1; 56.5-63.5	59.4; 55.8-62.8	28.9; 26.6-31.2	23.3; 20.3-26.4	34.4; 31.0-37.8
6-24 months	71.3; 67.8-74.5	66.9; 61.4-72.0	74.7; 70.2-78.8	28.5; 25.2-31.9	20.1; 15.8-24.9	35.1; 30.4-39.9
> 24 months	82.6; 76.6-87.6	80.2; 70.8-87.6	84.8; 76.4-91.0	28.4; 22.2-35.3	15.8; 9.1-24.7	40.2; 30.6-50.4

25-39 years old	60.4; 58.5-62.3	53.1; 50.2-56.0	66.1; 63.6-68.5	25.6; 24.0-27.4	15.1; 13.1-17.3	33.9; 31.4-36.4
< 6 months	46.4; 42.6-50.2	43.1; 37.7-48.5	49.6; 44.3-54.9	26.3; 23.0-29.7	19.6; 15.5-24.2	32.7; 27.8-37.8
6-24 months	61.8; 58.7-64.8	55.0; 49.9-60.0	66.2; 62.3-70.0	25.0; 22.3-27.9	13.5; 10.2-17.3	32.5; 28.7-36.4
> 24 months	69.4; 66.3-72.4	59.5; 54.6-64.3	77.6; 73.7-81.1	25.9; 23.1-28.9	13.0; 9.9-16.6	36.4; 32.2-40.8

40-64 years old	47.2; 45.2-49.2	43.0; 40.1-45.9	51.2; 48.4-54.1	19.8; 18.2-21.4	12.4; 10.5-14.4	26.8; 24.3-29.4
< 6 months	38.0; 33.1-43.1	38.8; 31.7-46.3	37.2; 30.5-44.4	17.8; 14.0-22.0	11.5; 7.2-17.0	23.7; 17.9-30.3
6-24 months	44.0; 40.3-47.7	39.1; 33.5-45.0	47.5; 42.6-52.4	21.7; 18.7-24.9	16.4; 12.4-21.2	25.4; 21.2-29.9
> 24 months	51.8; 49.0-54.5	45.8; 42.0-49.6	58.5; 54.4-62.4	19.4; 17.2-21.7	10.9; 8.7-13.5	28.9; 25.3-32.7

### 3.3 Risky alcohol drinking

According to the AUDIT-C, 24.8% of the sample were risky drinkers. Gender (p < 0.001) and age (p < 0.01) were significantly related to risky drinking, duration of unemployment was not. In total, the proportion for men was double the proportion for women (32.1% vs. 16.5%). Gender differences were found in all age groups and groups of unemployment. In addition, the 18-24 year olds had the highest proportions of risky drinkers (Table 2).

### 3.4 Overweight

Of the sample, 3.4% were underweight, 51.0% were normal weight, 30.1% were overweight, and 15.5% were obese. Gender (p < 0.001), age (p < 0.001) and duration of unemployment (p < 0.01) were significantly related to overweight/obesity (Table 3). Men were more often overweight/obese than women, in particular in both older groups. For men and women the proportions were higher with increasing age. There were higher proportions among the long-term unemployed compared to the short-term unemployed, more so for women and both younger groups. Overall, the highest proportion was found for 40-64 year old men.

**Table 3:** Proportions and 95% confidence intervals of overweight/obesity and low fruit and vegetable intake stratified by gender, age and months unemployed

	Overweight/obesity			Low fruit and vegetable intake		
	
	Total	Women	Men	Total	Women	Men
N	7,794	3,631	4,163	7,515	3,512	4,003
Total	45.6; 44.5-46.7	40.4; 38.8-42.0	50.1; 48.6-51.6	96.9; 96.5-97.3	96.0, 95.3-96.6	97.7; 97.1-98.1

18-24 years old	28.9; 27.2-30.7	26.7; 24.2-29.2	30.9; 28.5-33.5	96.9; 96.1-97.5	96.8; 95.7-97.8	96.9; 95.8-97.7
< 6 months	26.5; 24.3-28.7	22.9; 20.0-26.0	29.9; 26.8-33.2	96.8; 95.8-97.6	96.8; 95.3-97.9	96.8; 95.4-97.9
6-24 months	32.0; 28.7-35.5	32.7; 27.6-38.1	31.5; 27.1-36.2	97.1; 95.7-98.2	97.5; 95.2-98.9	96.8; 94.7-98.3
> 24 months	37.4; 30.8-44.4	38.1; 28.5-48.6	36.7; 27.7-46.5	96.0; 92.3-98.3	94.8; 88.3-98.3	97.2; 92.0-99.4

25-39 years old	43.1; 41.3-45.0	35.5; 32.8-38.3	49.0; 46.5-51.6	96.3; 95.5-97.0	94.9; 93.5-96.1	97.3; 96.4-98.1
< 6 months	34.8; 31.2-38.4	25.1; 20.6-30.0	44.0; 38.8-49.3	94.7; 92.7-96.2	93.2; 90.0-95.7	96.1; 93.5-97.8
6-24 months	41.8; 38.7-44.9	32.1; 27.5-37.0	47.9; 43.9-51.9	96.6; 95.3-97.6	95.7; 93.1-97.4	97.2; 95.6-98.4
> 24 months	51.2; 48.0-54.5	47.5; 42.6-52.4	54.3; 49.9-58.7	97.2; 95.9-98.1	95.7; 93.3-97.4	98.4; 96.9-99.3

40-64 years old	65.4; 63.5-67.2	58.8; 56.0-61.6	71.7; 69.1-74.1	97.5; 96.8-98.1	96.1; 94.9-97.2	98.9; 98.1-99.4
< 6 months	65.5; 60.6-70.3	58.1; 50.6-65.2	72.5; 65.8-78.6	97.6; 95.5-98.9	96.2; 92.3-98.4	99.0; 96.4-99.9
6-24 months	65.5; 61.9-68.9	53.4; 47.5-59.2	73.9; 69.4-78.0	98.0; 96.7-98.9	95.6; 92.6-97.6	99.8; 98.7-100.0
> 24 months	65.9; 63.2-68.4	62.1; 58.3-65.7	70.1; 66.3-73.7	97.2; 96.2-98.0	96.4; 94.7-97.6	98.2; 96.8-99.1

### 3.5 Low fruit and vegetable intake

Of the sample, 6.4% reported a fruit and vegetable intake of null portions per day, 35.5% one portion, 30.8% two portions, 18.4% three portions, 5.8% four portions, and 3.1% five or more portions. Thus, 96.9% of the sample consumed < 5 portions per day (Table 3). Gender (p < 0.001) and age (p < 0.05) were significantly related to low fruit and vegetable intake, duration of unemployment was not. In general, compared to women, (particularly older) men more often reported < 5 portions per day. Older men (40-64 year old) had a lower fruit and vegetable intake than younger men (18-24, 25-39 year olds).

### 3.6 Physical inactivity

Forty-three percent of the sample reported ≤ 30 minutes of every day physical activity, 59.6% reported < 1 hour of sports activity per week, and 28.3% were regarded as physically inactive. Gender (p < 0.001) and age (p < 0.1) were significantly related to physical inactivity, duration of unemployment was not (Table 4). Women, particularly the 18-24 year olds, were more often physically inactive than men. Both older age groups were more often physically inactive than the youngest group, particularly among men.

**Table 4:** Proportions and 95% confidence intervals of physical inactivity and illicit drug use stratified by gender, age and months unemployed

	Physical inactivity			Illicit drug use		
	
	Total	Women	Men	Total	Women	Men
N	7,539	3,518	4,021	7,473	3,493	3,980
Total	28.3; 27.3-29.3	30.2; 28.7-31.8	26.6; 25.2-28.0	7.6; 7.0-8.3	3.9; 3.3-4.6	10.9; 10.0-11.9

18-24 years old	25.1; 23.4-26.8	31.5; 28.9-34.2	19.3; 17.2-21.5	12.3; 11.0-13.6	6.9; 5.5-8.4	17.3; 15.3-19.4
< 6 months	26.0; 23.8-28.2	33.3; 30.0-36.7	18.8; 16.2-21.7	9.8; 8.4-11.4	5.0; 3.6-6.8	14.6; 12.2-17.3
6-24 months	24.9; 21.8-28.2	29.6; 24.7-34.9	21.3; 17.4-25.5	16.6; 14.0-19.5	10.6; 7.4-14.5	21.3; 17.4-25.6
> 24 months	18.8; 13.7-24.9	22.9; 15.0-32.6	15.1; 8.9-23.4	15.9; 11.2-21.7	6.3; 2.3-13.1	21.9; 14.4-31.0

25-39 years old	29.4; 27.6-31.2	29.9; 27.2-32.6	29.0; 26.7-31.4	9.3; 8.2-10.4	4.0; 2.9-5.3	13.4; 11.7-15.3
< 6 months	25.6; 22.4-29.0	27.9; 23.2-33.0	23.4; 19.1-28.1	7.2; 5.4-9.4	4.4; 2.5-7.2	9.9; 7.0-13.5
6-24 months	31.7; 28.9-34.7	33.5; 28.8-38.4	30.6; 27.0-34.4	8.9; 7.2-10.8	3.6; 2.0-5.9	12.3; 9.7-15.1
> 24 months	29.7; 26.7-32.7	28.1; 23.8-32.6	31.0; 27.0-35.2	11.3; 9.3-13.5	3.9; 2.2-6.2	17.3; 14.1-20.9

40-64 years old	30.5; 28.7-32.4	29.3; 26.7-32.0	31.7; 29.1-34.3	0.9; 0.6-1.4	0.8; 0.4-1.5	1.1; 0.6-1.8
< 6 months	32.5; 27.9-37.5	28.8; 22.4-35.9	36.0; 29.3-43.2	1.6; 0.6-3.4	0.5; 0.0-3.0	2.6; 0.8-5.9
6-24 months	31.2; 27.8-34.7	28.1; 23.1-33.6	33.3; 28.8-38.1	0.4; 0.1-1.2	0.3; 0.0-1.9	0.5; 0.1-1.7
> 24 months	29.5; 27.1-32.1	30.0; 26.6-33.6	29.0; 25.5-32.8	1.0; 0.5-1.7	1.0; 0.4-2.1	1.0; 0.4-2.1

### 3.7 Illicit drug use

Eight percent of the total sample had used illicit drugs in the past 12 months (Table 4). Gender (p < 0.001), age (p < 0.001) and duration of unemployment (p < 0.01) were significantly related to illicit drug use. Men used illicit drugs about two to three times more often than women (3.9% vs. 10.9%), and the 18-24 year olds had higher proportions of drug use than both older groups. In particular among the 18-24 year olds, the medium-unemployed women and men had higher proportions of drug use than the short-term unemployed. Overall, the highest proportions were found for medium- and long-term unemployed 18-24 year old men (21.3%, 21.9%).

### 3.8 Total number of health risk factors

Among all participants, 0.5% (n = 40) had none of the six health risk factors investigated, 13.5% (n = 998) had one, 33.6% (n = 2,494) had two, 33.4% (n = 2,481) had three, 15.1% (n = 1,119) had four, 3.6% (n = 265) had five, and 0.3% (n = 21) had all six health risk factors. Thus, 52.4% of the participants had ≥ 3 health risk factors (Table 5). Gender (p < 0.001), age (p < 0.05) and duration of unemployment (p < 0.001) were significantly related to having ≥ 3 health risk factors. Men more often than women had ≥ 3 health risk factors. Among men, the 25-39 and the 40-64 year old had higher proportions of ≥ 3 health risk factors than the 18-24 year olds. In addition, with increasing duration of unemployment, the proportions of having ≥ 3 health risk factors increased, particularly among the 18-24 and 25-39 year olds. Overall, the highest proportion of ≥ 3 health risk factors was found for long-term unemployed 25-39 year old men (73.4%).

**Table 5:** Proportions and 95% confidence intervals of having ≥ 3 health risk factors stratified by gender, age and months unemployed

	Total	Women	Men
N	7,418	3,473	3,945
Total	52.4; 51.2-53.5	43.8; 42.1-45.5	60.0; 58.4-61.5

18-24 years old	50.6; 48.6-52.6	46.4; 43.6-49.3	54.4; 51.6-57.1
< 6 months	46.9, 44.4-49.4	44.8; 41.2-48.3	49.0; 45.5-52.6
6-24 months	56.1; 52.4-59.7	49.8; 44.2-55.5	61.0; 56.0-65.8
> 24 months	59.4; 52.2-66.3	48.4; 38.0-58.9	69.6; 59.7-78.3

25-39 years old	53.8; 51.9-55.8	41.6; 38.8-44.6	63.3; 60.8-65.8
< 6 months	41.8; 38.1-45.6	33.8; 28.8-39.2	49.4; 44.1-54.8
6-24 months	54.6; 51.5-57.8	41.5; 36.5-46.5	63.1; 59.1-67.0
> 24 months	62.1; 58.9-65.3	48.3; 43.3-53.3	73.4; 69.3-77.2

40-64 years old	52.7; 50.7-54.8	43.2; 40.3-46.1	61.9, 59.1-64.6
< 6 months	49.3; 44.2-54.5	39.3; 32.2-46.8	58.8; 51.5-65.8
6-24 months	52.6; 48.8-56.3	41.4; 35.7-47.3	60.5; 55.6-65.3
> 24 months	53.9, 51.1-56.6	45.1; 41.3-48.9	63.9; 59.9-67.7

### 3.9 Predicting self-rated health

Of all participants, 9.3% reported excellent health, 22.6% very good health, 50.6% good health, 12.9% fair health, and 4.6% poor health. For the analyses, the sample was split in two groups: excellent/very good health vs. good/fair/poor health. This procedure most closely resulted in two groups of roughly equal size: 31.9% with better health and 68.1% with lower health. Multivariate logistic regression analyses revealed that overweight and physically inactive women and men had significantly increased odds of lower self-rated health (Table 6). While among women, low fruit and vegetable intake was significantly related to lower self-rated health, and illicit drug use was not, the reverse was true for men. After conducting the same prediction equation with gender as a covariate among the total sample, tobacco smokers and risky drinkers also showed increased odds of lower self-rated health. A final logistic regression analysis with the total number of health risk factors as the predictor revealed an OR of 1.34 (95% CI 1.28-1.42).

**Table 6:** Multivariate logistic regressions to predict low self-rated health*

		Women (n = 3,441)		Men (n = 3,908)		Total sample (n = 7,349)	
		
Variables		OR	95% CI	OR	95% CI	OR	95% CI
Tobacco smoking (No)	Yes	1.13	0.96-1.34	1.14	0.97-1.33	1.15	1.02-1.29
Risky drinking (No)	Yes	1.22	0.98-1.52	1.15	0.99-1.35	1.17	1.03-1.33
Overweight (No)	Yes	1.68	1.41-2.00	1.63	1.40-1.89	1.67	1.49-1.87
Low fruit and vegetable intake (No)	Yes	1.58	1.09-2.28	1.41	0.90-2.19	1.55	1.16-2.05
Physical inactivity (No)	Yes	1.47	1.23-1.76	1.78	1.51-2.11	1.66	1.47-1.88
Illicit drug use (No)	Yes	0.92	0.62-1.37	1.29	1.02-1.62	1.14	0.93-1.38
Gender (Male)	Female	-	-	-	-	1.67	1.50-1.87
Age group (18-24)	25-39 years	0.97	0.79-1.20	1.14	0.96-1.36	1.06	0.93-1.22
	40-64 years	1.60	1.19-2.15	2.72	2.08-3.55	2.17	1.78-2.64
Life-time unemployment (< 6 months)	6-24 months	1.16	0.96-1.41	1.45	1.22-1.73	1.31	1.15-1.49
	> 24 months	1.93	1.53-2.44	1.92	1.56-2.37	1.88	1.61-2.19
School (> 11 years)	< 10 years	1.44	1.10-1.87	1.60	1.27-2.00	1.54	1.30-1.82
	10-11 years	1.43	1.17-1.74	1.31	1.08-1.59	1.35	1.18-1.55
Family status (Single)	Married	1.05	0.83-1.34	1.64	1.29-2.08	1.29	1.09-1.53
	Divorced	0.99	0.73-1.35	1.19	0.87-1.62	1.09	0.87-1.35
	Widowed	1.13	0.54-2.34	4.11	0.94-18.03	1.49	0.78-2.82

Notes: *Health was rated as good, fair or poor as opposed to excellent or very good.

OR = odds ratio, 95% CI = 95% confidence interval.

Brackets indicate reference category.

## 4. Discussion

This study revealed four main findings: Firstly, very high proportions of individuals with health risk behaviors were found, and associations with self-rated health were confirmed. Secondly, the data confirmed that socially less well-off include particularly high proportions of individuals with health risk behaviors. Thirdly, the data revealed a higher number of health risks for long-term unemployed than for short-term unemployed individuals. And fourthly, the findings indicate that approaching unemployed individuals at job agencies may be a promising approach for prevention efforts.

In comparison to the adult general population, job-seekers recruited at job agencies live unhealthier in various ways. Among them, a larger proportion of individuals smoke (57.7% vs. 30% [16]), do not eat the recommended 5+ portions of fruit and vegetables per day (96.9% vs. 60-75% [17]), do sports less than one hour per week (59.6% vs. 47.7% [18]), and use illicit drugs (7.6% vs. 5.1% [19]). However, we have to bear in mind that the reported discrepancies may in part be explained by different methodology and sample selection effects, causing e.g. different mean ages in our study compared to the reference data.

As expected, all six health risk factors investigated were related to self-rated health, which serves as a reliable proxy of health [14]. Partly, these relationships differed by gender. In general, overweight and physically inactive individuals reported lower health. In addition, male illicit drug users and females with low fruit and vegetable intake also reported lower health. Those women and men, who were smoking or drinking at risky levels had slightly increased odds of lower self-rated health. However, the larger total sample with increased power to detect small effects was needed to confirm that these two health risk factors were significantly related to self-rated health. Furthermore, our findings accentuate the strong relationship between the number of health risk factors and one's own health. With each additional health-risk factor the odds of lower self-rated health increased by 34%. However, longitudinal data are needed to test causal relationships (as reported below).

Gender and age differences were found for all health risk factors. Briefly, five of the six health risk factors were more prevalent among men than women, and physical inactivity was more prevalent among women than men. All three substance use related health risk factors were more prevalent among younger than among older individuals. The reverse was true for factors related to nutrition and physical inactivity. In accordance with previous findings [20], men more often had three or more health risk factors than women, and these proportions were higher with increasing age.

In line with previous findings [7], the longer the duration of unemployment, the higher the proportions of individuals with health risk behaviors. Among the long-term unemployed individuals particularly high proportions of current smokers, overweight/obese individuals and current drug users were found. Moreover, having three or more health risk factors was more common among long-term than among short-term unemployed individuals.

Several implications for behavioral interventions to increase health among job-seekers may be derived from our findings: 1) As the vast majority of job-seekers (86.0%) exhibit more than one health risk factor, multiple behaviour change interventions may be adequate. 2) For primary prevention purposes these interventions should be offered to all job-seekers with particular risk factors, and not only to those with the most severe combination of health risk factors. E.g. although long-term unemployed job-seekers have the highest proportions of risk factors, short- and medium term unemployed job-seekers also have increased proportions in comparison to the general population. And 3) interventions targeting on substance use are required particularly for younger job-seekers; and interventions targeting on factors related to nutrition and physical activity are required particularly for older job-seekers.

Some limitations and peculiarities of the study must be mentioned. Firstly, we used cross-sectional analyses to describe the population of job-seekers reached at job agencies. We cannot draw any conclusion regarding causal relationships, e.g. between health risk factors and self-rated health. Clearly, longitudinal data are required to test "what comes first?"-questions such as: Is health impaired because of physical inactivity or are people physically inactive because of impaired health? Secondly, we solely relied on self-report. Future research needs to investigate 'hard' health outcomes, such as biological measures, cardiovascular events or mortality. Thirdly, as we had to create a brief questionnaire to increase compliance among potential study participants, our assessment of physical inactivity may have been somewhat inaccurate. We only assessed one aspect of every day physical activity namely commuting, and neglected others such as housework activities, care of children and occupational activities, which may have resulted in an underestimation of every day physical activity. In contrast, we may have overestimated sports activity as we did not ask about the intensity of sports activity. These aspects should be assessed in future studies. Fourthly, as our aim was to investigate job-seekers that can be approached at job agencies, our sample included unemployed individuals, but also not yet unemployed individuals. Thus, all proportions reported have been stratified by duration of unemployment. And fifthly, a sample selection bias may have occurred as we have drawn our sample from one single area of Germany, which is characterized by an elevated unemployment rate in comparison to Germany in general (12.5% vs. 7.0%) [21]. Some of our findings may not apply to job-seekers in other areas within and beyond Germany. Unfortunately, no comparable data from other areas were available.

## 5. Conclusion

Job agencies appear to provide a suitable setting to contact individuals pro-actively for health behavior change interventions. Although, many job-seekers may have other existential issues when presenting at job-agencies, the fairly high participation rate in our study (79.8%) is encouraging. Consequentially: 1) A large part of individuals with health risk behaviors may be contacted, and high proportions of participants among the target population of unemployed people may be achieved, particularly among the long-term unemployed people, who appear to have the highest health risks. 2) As high proportions of participants may be recruited in a short period of time, such an approach might turn out as cost-saving. 3) Individuals who do not intend to change their behavior may be included. 4) Pro-active recruitment of job-seekers at job-agencies, with the aim to contact and motivate each individual of the target group to participate in a prevention program, may result in a higher number of job-seekers than would normally be reached. Particularly those hard to reach, not motivated to participate or those with the highest health risks may be accessed. For example, the rate of current smokers in our sample was 7.2% higher than the rate found among job-seekers recruited through a German general population survey [4]. 5) Pro-active intervention efforts may be implemented quite efficiently, also when multiple contacts are required, because the job-seekers are required to be present on a regular basis. And 6) Participants and job agencies could benefit from healthier life-styles as these are related to better health and higher chances of placement [22,23].

Health behavior change interventions such as computer expert system technology or motivational interviewing can be effective in large populations [24,25]. However, the efficacy of such interventions when provided at job agencies must be investigated still.

## Competing interests

The authors declare that they have no competing interests.

## Authors' contributions

JFA designed the study, performed the statistical analyses and drafted the manuscript. BG and UJ participated in the design of the study. BG helped to perform the statistical analyses. BG and ST participated in the coordination and recruitment of the study sample. All authors read and approved the final manuscript.

## Pre-publication history

The pre-publication history for this paper can be accessed here:

http://www.biomedcentral.com/1471-2458/11/659/prepub
